# Examining the feasibility and utility of heart rate variability on intervention outcomes targeting emotion regulation in autism: a brief report

**DOI:** 10.1038/s41598-024-66084-z

**Published:** 2024-07-04

**Authors:** Debra L. Reisinger, Matthew S. Goodwin, Paul S. Horn, Lauren M. Schmitt, Marika C. Coffman, Rebecca C. Shaffer

**Affiliations:** 1 Division of Behavioral Medicine and Clinical Psychology, Cincinnati Children’s Hospital Medical Center, Cincinnati, OH USA; 2 Department of Pediatrics, University of Cincinnati College of Medicine, Cincinnati, OH USA; 3 Department of Health Sciences, Northeastern University, Boston, MA USA; 4 Division of Neurology, Cincinnati Children’s Hospital Medical Center, Cincinnati, OH USA; 5 Duke Center for Autism and Brain Development, Department of Psychiatry and Behavioral Sciences, Duke University, Durham, NC USA

**Keywords:** Autism, Intervention, Heart rate variability, Emotion regulation, Interventional cardiology, Human behaviour

## Abstract

Autistic youth experience several behavioral and emotional characteristics that can predispose them to emotion dysregulation (ED). Current literature examining ED in autism spectrum disorder (ASD) is limited to parent- and self-reported measures, indicating a need for biological or physiological methods to better assess emotion regulation in ASD. Utilizing the autonomic nervous system, specifically heart rate variability (HRV), may be a promising method to objectively measure ED in ASD, given it is one of the body’s primary means of regulating physiological arousal. Our pilot study is one of the first to examine the feasibility, utility, and construct validity of HRV along with clinical measures within an intervention targeting ED-specific symptoms in ASD. Participants included 30 autistic youth ages 8–17 years who participated in the pilot study of *Regulating Together*, a group-based intervention targeting emotion regulation. We demonstrate HRV is feasible, demonstrates adequate test–retest reliability, and is complimentary to clinician- and parent-reported measures. Our preliminary findings also point to certain HRV profiles being indicative of long-term outcomes after receiving treatment. HRV may be a useful, objective tool in determining differential needs of long-term follow-up care for treatment maintenance at screening or baseline stages.

## Introduction

Emotion regulation (ER) involves the ability to monitor and modify one’s level of arousal and reactivity to engage in adaptive behavior that is goal-directed^1–3^. It is a complex, multifaceted, and interactive process involving neurobiology, peripheral physiology, cognition, behavior, affect, and context. Multiple characteristics associated with autism spectrum disorder (ASD) can predispose individuals to maladaptive ER or Emotion Dysregulation (ED), including alexithymia (e.g., difficulty verbalizing emotions), cognitive and behavioral rigidity, poor perspective taking, limited social insight, sensory processing difficulties, impulsivity, and impaired problem-solving^2,4,5^. Inadequate ER skills have been linked to poorer outcomes in autistic youth with co-occurring ED in comparison to those without co-occurring ED^6–9^. Most of the work examining ER in ASD utilizes self- or parent-reported measures and behavioral observations to assess ER^10^. However, given ER's complex, multifaceted nature, researchers have advocated for using biological or physiological methods to evaluate ER in ASD^11,12^. The autonomic nervous system (ANS) is a promising, objective way to assess children’s emotional functioning as it is one of the body’s primary means of regulating physiological arousal. This may be especially true for autistic individuals who are minimally verbal or alexithymic. While the literature has begun to identify differences in the ANS in relation to ER, a gap remains utilizing and examining physiological methods within treatments targeting ER in autism.

Physiological emotional arousal originates from the ANS, which is comprised of both sympathetic and parasympathetic divisions. The sympathetic nervous system (SNS) is active during stressful situations, and the parasympathetic nervous system (PNS) is predominant during rest, relaxation, and recovery^13,14^. Heart rate variability (HRV) is a non-invasive, feasible, and objective indicator of autonomic activity, particularly parasympathetic influences on cardiac recovery^14^. HRV is the variation in time between successive heartbeats or the interval between sequential R–R peaks in the QRS complex. When the SNS is activated, it shortens R–R intervals. In contrast, the PNS prolongs the time between R–R intervals. Previous work theorizes that HRV yields information about autonomic adaptability and represents the capacity for regulated emotional responding, serving as an index of ER^15–19^. More specifically, higher resting HRV has been associated with increased ER and use of appropriate coping strategies, whereas lower resting HRV is related to ED and less constructive strategies^20–22^.

Youth who can regulate their physiological arousal display fewer problem behaviors^23,24^. In contrast, studies of psychiatric disorders often characterized by ED, such as bipolar disorder and antisocial behavior, report a strong association between increased physiological arousal and symptomatology^25–27^. Similarly, physiological arousal of the ANS is consistently implicated in the presence of aggressive behavior in autistic youth as well as typically developing youth^28^. The earliest descriptions of ASD highlight atypical physiological states and offer a theory that externalizing behaviors are functionally related to homeostatic regulation^29–32^. While significant heterogeneity in ASD exists, recent work demonstrates that atypical autonomic reactivity is a common feature^33–35^ and associated with maladaptive behavior^36,37^.

Within the ASD literature, altered autonomic responses may be one of the mechanisms of behavioral dysregulation^38^. Similar to typically developing individuals, ASD and other psychiatric conditions, including depression, anxiety, and psychosis associated with poor ER, demonstrate lower HRV^39–42^, whereas high HRV is associated with better ER^43^. A recent meta-analysis concluded that autistic individuals have altered PNS activity as indicated by lower HRV during social stress, social debriefing, and cognitive tasks in comparison to controls^40^. Since ER is hypothesized to be a transdiagnostic mechanism underlying several mental health disorders, including ASD^1,44,45^, there is clinical utility in examining the relationship between HRV and ER in individuals with autism. For example, assessing HRV in ASD as a marker of ED may provide an advantage to quantifying dysregulated responses that are missing in other measures (e.g., caregiver or self-report) or complicated by confounding factors (e.g., limited language, lack of behavioral responses)^11^. For example, Baker et al.^46^ found baseline respiratory sinus arrhythmia (RSA), the naturally occurring variation in HR during the breathing cycle, less useful as a dysregulation index in ASD. However, higher RSA reactivity occurring during a challenging task designed to elicit physiological arousal may predict an underlying risk for observed ED in ASD^46^. Additionally, recent work by Goodwin et al.^47^ demonstrates continuous HRV data can be used to predict aggressive behavior 1–3 min before it occurs in minimally verbal and severely impacted autistic youth^48^. While this literature provides evidence for HRV being an index of ER, HRV has not yet been examined or described within the context of targeted treatment for ER in ASD.

The present paper addresses these limitations by examining the feasibility, reliability, and utility of collecting cardiovascular data as a potential biomarker in relation to treatment change in a pilot intervention trial focusing on ED in ASD^49^. Within ASD intervention research, there are notable limitations in measuring treatment progress based solely on caregiver and clinician reports. While there has been some research examining the relationship between HRV and ER in autistic youth^50,51^, limited work has been conducted examining the relationship between HRV and clinical outcomes after targeted ER treatment. Given the promising results of our pilot study based on our caregiver- and clinician-rated clinical outcome measures^49^ and exploratory findings on the feasibility of using an objective behavioral measure of cognitive flexibility^52^, in the present study, we aimed to (1) examine the feasibility and construct validity of utilizing HRV within a treatment trial as a methodology for treatment change and (2) explore the relationships between HRV and specific outcome measures associated with improvement over time after completing a targeted ER intervention. We hypothesized that HRV would be feasible to collect in autistic youth with co-occurring ED. We also hypothesized that HRV will demonstrate construct validity with several clinical outcome measures. Lastly, although exploratory, we hypothesized that baseline HRV and HRV changes across time would predict clinical outcomes post-treatment.

## Methods

### Participants

The present study included 30 participants (83.3% male) with a DSM-5 diagnosis of ASD ages 8–17 years (*M* = 12.05, *SD* = 2.62) who participated in the pilot within-subjects design of the Regulating Together^49^ intervention trial across five different groups. The sample was primarily non-Hispanic (90%) and White (90%), with attention-deficit/hyperactivity disorder (ADHD) being the most common co-occurring diagnosis (73.3%). At least one psychotropic medication was taken by 66.7% (N = 20) of participants. Sociodemographic variables collected included household income, highest level of parental education, and the number of adults and children living at home. During screening, all participants completed the Wechsler Abbreviated Scales of Intelligence, Second Edition (WASI-II)^53^ to assess cognitive ability. All participants completed the Autism Diagnostic Observation Schedule, Second Edition (ADOS-2)^54^ to confirm their ASD diagnosis, of which six participants completed the ADOS-2 outside of our clinic within the past 6 months prior to their screening visit; their ASD diagnosis was confirmed based on their diagnostic report, but scores were not available. Participants were excluded from the intervention trial if they did not meet DSM-5 criteria for ASD, were not able to communicate using complex speech (e.g., occasional utterances with two or more clauses), did not have at least one caregiver available to participate in the study, English was not their or their caregiver’s primary language, or had an IQ < 60. Participant inclusion criteria included a score ≥ 10 on either the Irritability or Hyperactivity subscale of the Aberrant Behavior Checklist, Second Edition^55^. This study was approved by the Cincinnati Children’s Hospital Medical Center Institution Review Board (IRB) and was conducted in accordance with all relevant guidelines and standards. All caregivers provided informed written consent, and participant assent was obtained orally when appropriate. See Table 1 for detailed participant descriptive statistics and sociodemographic information.

**Table 1:** Participant descriptive statistics.

Variable	*n*	%	Mean	*SD*	Min	Max
Participant age (years)	30		12.50	2.62	8.19	17.86
Participant race						
White	27	90.00				
Two or more races	2	6.67				
Asian	1	3.33				
Ethnicity						
Hispanic	3	10.00				
Non-Hispanic	27	90.00				
Co-occurring diagnoses						
ADHD	22	73.3				
Anxiety disorder	15	50.00				
Depression	7	23.33				
Intermittent explosive disorder	5	16.67				
Insomnia	5	16.67				
Obsessive–compulsive disorder	4	13.33				
Oppositional defiant disorder	2	6.67				
Full scale IQ	30		100.13	18.98	47.00	137.00
ADOS-2 SA CSS	26		7.42	1.65	3.00	10.00
ADOS-2 RRB CSS	26		6.69	2.15	1.00	10.00
ADOS-2 Total CSS	26		7.19	1.47	3.00	10.00
ABC-2 Irritability	30		22.50	10.37	8.00	50.00
ABC-2 Hyperactivity	29		12.10	7.68	2.00	28.00
Household Income						
< $20,000	1	3.33				
$20,001–40,000	3	10.00				
$40,001–60,000	5	16.67				
$60,001–90,000	3	10.00				
> $90,000	18	60.00				
Parental education (highest level)						
Some high school	1	3.33				
High school graduate or GED	3	10.00				
Some college/post-high school or 2-year degree	6	20.00				
College graduate	10	33.33				
Advanced graduate/professional degree	10	33.33				
Number of Adults in the Home	26		2.04	0.60	1.00	4.00
Number of Children in the Home	25		2.32	1.41	1.00	6.00

ADOS-2, Autism Diagnostic Observation Schedule; Second Edition; SA, Social Affect; CSS, Calibrated severity score; RRB, Restricted; repetitive behavior; ABC-2, Aberrant Behavior Checklist; Second Edition; GED, General Education Development; ADHD, attention deficit/hyperactivity disorder.

### Intervention

**Figure 1:**
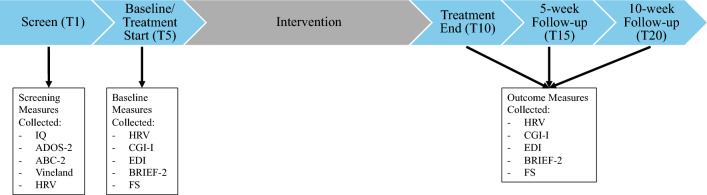
Timeline of data collection from Baseline to 10-week post-intervention follow-up, including the measures that were collected. IQ, Intelligence Quotient; ADOS-2, Autism Diagnostic Observation Schedule; Second Edition; ABC-2, Aberrant Behavior Checklist; Second Edition; HRV, Heart Rate Variability; CGI-I, Clinical Global Impression Scale—Improvement; EDI, Emotion Dysregulation Inventory; BRIEF-2, Behavior Rating Inventory of Executive Functioning; Second Edition; FS, Flexibility Scale—Revised.

Regulating Together^49^ is a 10-session, 5-week group-based ER intervention for autistic children and adolescents and their caregivers. See Shaffer et al.^49^ for detailed information about the curricula. Participants completed a 5-week control lead-in period and a 5-week active treatment period with follow-up visits at 5- and 10-weeks post-intervention completion. Participants completed 5 study visits in total, where baseline and outcome data were collected, including HRV: Screen (T1), Baseline/Treatment Start (T5), Treatment End (T10), Treatment Follow-up 1 (T15), and Treatment Follow-up 2 (T20) (Fig. 1).

### Cardiovascular data collection and processing

#### Data collection

**Figure 2:**
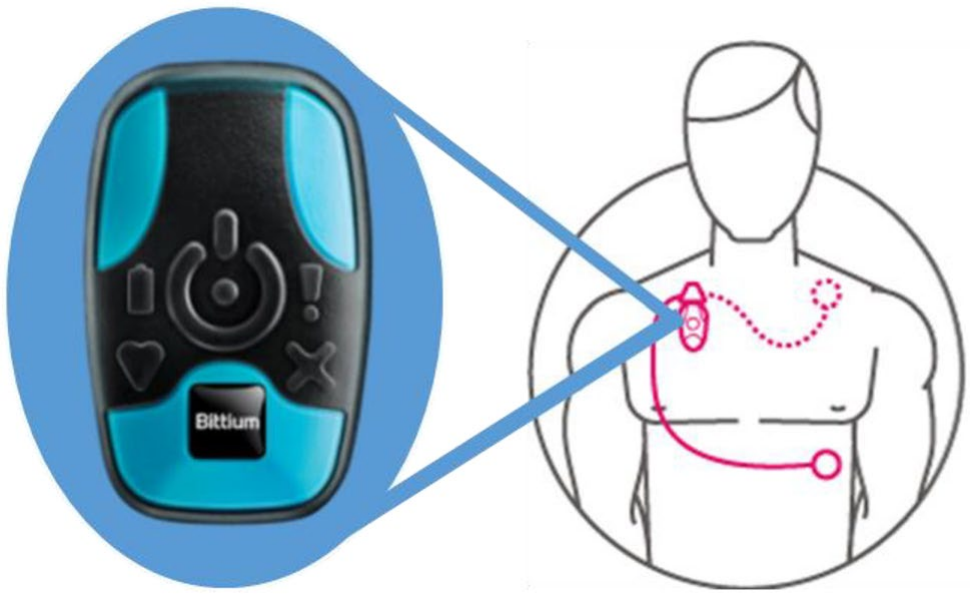
Bittium 180° eMotion Faros™ Cardiac Monitor.

**Figure 3:**
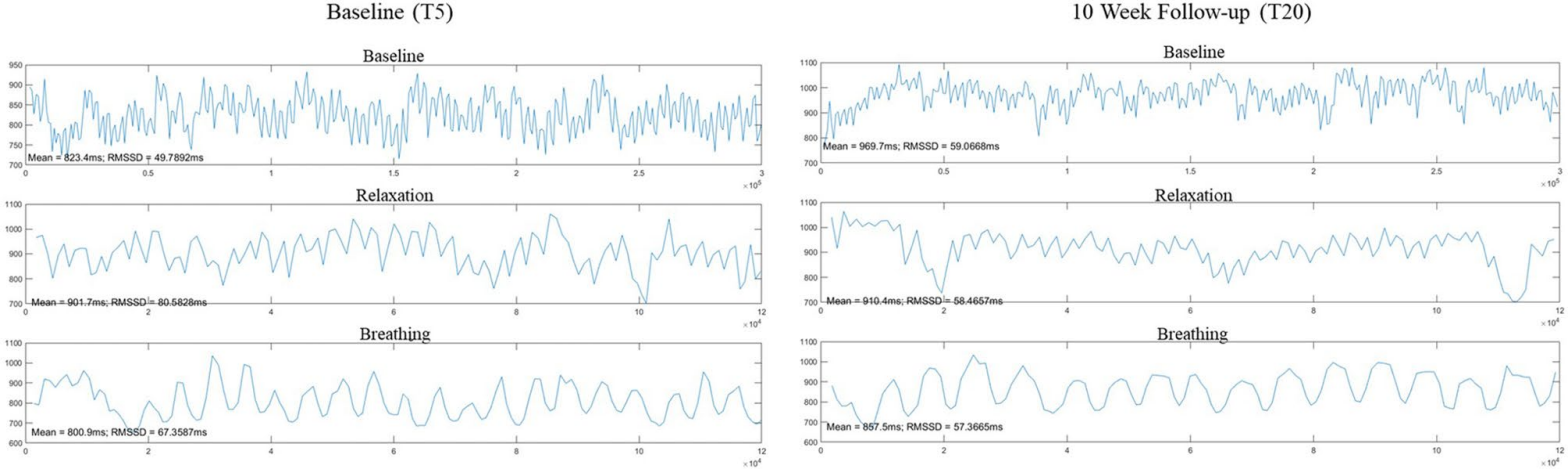
Example HRV collection by task utilizing RMSSD for one 8–12-year-old participant at Baseline (T5) and their 10-week follow-up visit (T20).

Cardiovascular data was collected wirelessly using the Bittium 180° eMotion Faros™ Cardiac Monitor at a sampling rate of 1000 Hz. The 180° Faros monitor is a wearable, portable, externally applied 1-channel electrocardiograph (ECG) recorder for ECG and HRV (R–R interval) data measurement. For the present study, we utilized the cable with three electrode placements to allow for the best skin contact during the measurement process (Fig. 2). The reference (ground) electrode was placed below the left clavicle, one electrode was placed below the right clavicle, and one electrode was placed on the left side of the chest on the ribs. Our dependent variable included the root mean square difference of successive R-R intervals (RMSSD). RMSSD is a standard and reliable time domain measure of HRV, is recommended to measure short-term components of HRV, and is suggested to reflect cardiac vagal control predominantly^13^. See Fig. 3 for an example of one 8–12-year-old participant who completed the study.

HRV data were continuously measured across three different tasks: Baseline (5 min), Relaxation (2 min), and Breathing (2 min). To be consistent between participants, tasks were administered in a fixed sequence to maintain any carryover effects across activities. Data were aimed to be collected at all baseline and post-intervention visits. HRV was added during the pilot intervention trial, resulting in some missing data at the T1 and T5 visits. Additionally, COVID-19 occurred during the end of the pilot intervention study, resulting in HRV data missing for 18 participants at their outcome visits and 8 at their T1 visit. For participants who had HRV data at T1 only, we used their T1 data (n = 8) for T5, given that the intervention had not yet started. See Table 2 for a breakdown of HRV data collected at each time point, including the initial and final samples used for the present study.

**Table 2:** Number of HRV collections by timepoint.

	Visit timepoint				
Variable	T1	T5	T10	T15	T20
Initial data collected	18	20	5	2	9
Final sample	–	28	5	2	9

#### Data processing

Task recordings were set at 2 min for the sake of expedience/compliance. Specifically, since this was a pilot trial of HRV within the study, our team had to balance getting sufficient, appropriate data with protocol length and participant needs/tolerability. This is below the recommended recording time^13^ and comes with component risks^56^. However, the strong influence of slower respiratory cycles establish autonomic control immediately^57^; the coordinated cardiovascular response to respiration is parasympathetically mediated, and the relevant cholinergic transmission time is ~ 300 ms. Thus, a commonly used rule is that recordings must display 10 cycles of interest, which gives 2-min recordings (i.e., 120 s) resolution down to ~ 0.08 Hz, which contains the unconstrained breathing frequencies of interest. Measurement of HRV in other contexts frequently occurs over shorter intervals where autonomic modulation is expected to be substantial (e.g., acute and chronic recovery from athletic training). Frequencies below 0.05 Hz, which are unlikely to contain meaningful frequency information, were censored at all points.

Participant Baseline, Relaxation, and Breathing data were analyzed in the time domain by standard metrics (Mean IBI, RMSSD) and in the frequency domain by Lomb-Scargle Periodogram (LSP)^58,59^. Analysis by LSP requires no resampling and preserves isolated frequency peaks without smoothing, necessary to establish the center of provoked respiratory frequencies. Frequencies were calculated in 500 bins (0.001–0.5 Hz at 0.001 Hz intervals).

Relevant sections were isolated from longer recordings by timestamp, and beat-to-beat intervals were calculated from R-peaks detected with R-DECO^60^ (5 Hz high-pass filter; peak detection by cross-correlation). Some Baseline data were censored due to motion artifacts being present at the start of recording (n = 10, max data loss = 14 s). Two recordings displayed single atrial premature complexes (APCs), which we corrected using linear interpolation. No data was lost, and overall non-automated correction was minimal.

#### Procedures

As described above, at each assessment time point (T1, T5, T10, T15, T20), HRV data were collected in combination with a battery of screening or outcome measures. Prior to the start of HRV measurement, the 180° Faros monitor was synchronized with a laptop which was also recorded via video camera and used to track the duration of HRV during each activity for post-processing. Participants were given visuals to demonstrate where the placement of the electrodes occurs. A visual schedule for the entire HRV paradigm (in addition to their study visit visual schedule) was also offered. We also allowed participants to see the HRV monitor and try wearing the electrodes on their hands, and/or the examiners modeled wearing the device for the participants. The 180° Faros monitor was then placed on the participant's chest, as shown in Fig. 1. Participants were then instructed to watch one of 25-min baseline videos randomly selected at their T1 and then alternated at their subsequent visits. The baseline videos were chosen due to their calm and neutral content (two episodes from *Sarah & Duck)*. Since HRV data was collected across multiple time points, the baseline videos were alternated in a fixed sequence across participants and time points. After watching the baseline video, participants were provided a basket with various relaxation materials (e.g., markers, paper, expanding ball, liquid motion timer, infinity cube, squishy balls). Participants were instructed to spend a few minutes (2 min) relaxing however they chose, and the materials were available if they would like to use them. After 2 min passed, the relaxation materials were removed, and the participants were provided with two breathing visuals (Figure 8 and Triangle Breathing). They were instructed to spend a few minutes breathing and could choose to do so independently or use the visuals provided. The task was concluded after 2 min, and the HR monitor was removed. The Breathing and Relaxation tasks were chosen using key components of the Regulating Together^49^ curriculum, where participants were taught to engage in deep breathing using visual stimuli and other relaxation techniques. Participants transitioned between each HRV task rather quickly, wherein roughly 30-s to 1-min breaks between tasks occurred to switch out materials and get the next task started. The HRV paradigm took approximately 12 min on average for each participant from start to finish.

### Outcome measures

#### Emotion dysregulation inventory

The Emotion Dysregulation Inventory (EDI)^61,62^ is a caregiver-rated measure evaluating ED and was administered across all visit time points. This measure includes the Reactivity subscale (EDI-R), which measures regulated negative emotional responses, and the Dysphoria subscale (EDI-D), which measures poor uptake of positive emotions and lack of motivation. Raw scores are converted to Theta scores with a mean of 0 and an SD of 1 (similar to T-scores using a mean of 50 and an SD of 10). The EDI is a well-established change-sensitive measure of ED, with the Reactivity subscale demonstrating an internal consistency of 0.97 and the Dysphoria subscale demonstrating an internal consistency of 0.90^63^. For the purposes of the present study, we utilized EDI-R as a primary outcome measure and EDI-D as a secondary outcome measure.

#### Aberrant behavior checklist, second edition

The Aberrant Behavior Checklist, Second Edition (ABC-2)^55^ is a caregiver-rated 58-item scale targeting behavioral difficulties commonly seen in individuals with developmental disabilities. The scale comprises five subscales: Irritability, Social/Withdrawal, Lethargy, Stereotypy, Hyperactivity, and Inappropriate Speech. For the present study, we focused on the Irritability (ABC-I) subscale as it was an outcome measure used in the intervention study. Caregivers rated the severity of their child’s behavior using a 4-point Likert scale (0 = not a problem to 3 = the problem is severe in degree).

#### Clinical global impression scale—improvement

The Clinical Global Impression Scale – Improvement (CGI-I)^64^ was utilized as a clinician-rated outcome measure to assess response to treatment related to ED. The CGI-I was completed by a trained, independent clinician at T5, T10, T15, and T20 visits. The CGI provides a qualitative measure of treatment response using a scale of 1–7 (1 = very much improved; 2 = much improved; 3 = minimally improved; 4 = no change; 5 = minimally worse; 6 = much worse; 7 = much worse). Training for raters was conducted using gold-standard vignettes. Regular reliability training was conducted for all raters.

#### Behavioral rating inventory of executive functioning, second edition

The Behavioral Rating Inventory of Executive Functioning, Second Edition (BRIEF-2)^65^ is a caregiver-rated measure that includes 86 items measuring executive functioning skills in youth ages 5–18 years. Caregivers completed BRIEF-2 at all study visits. The measure includes 10 clinical scales across three index scores, including the Cognitive Regulation Index (CRI), Behavioral Regulation Index (BRI), and emotion regulation index (ERI). Raw scores are converted into T-scores across clinical and index scales. T-scores on the BRIEF-2 ERI were utilized in the present study.

#### Flexibility scale—revised

The Flexibility Scale—Revised (FS) is a non-normed caregiver report measure that includes 27 items targeting dimensions of flexibility in youth with ASD^66^. It provides raw scores across five subscales: Social Flexibility (range 0–15), Routines and Rituals (range 0–15), Transitions/Change (range 0–21), Special Interests (range 0–18), and Generativity (range 0–12) along with a FS Total Score. Items are rated on a 4-point Likert scale ranging from 0 (no) to 3 (always). Higher scores are indicative of greater difficulties with flexibility. Previous concerns documented by the authors suggest the Generativity subscale does not correlate highly with the other subscales of flexibility or other measures of executive function, but rather social and verbal symptoms of ASD. Therefore, we did not include the Generativity subscale in our analyses. Internal consistency within scales is adequate with correlations ranging from 0.75 to 0.91^66^. The present study utilized raw scores on the FS Total, Social Flexibility, and Transitions/Change subscales.

### Data analysis

All analyses were completed in SAS® 9.4 (SAS Institute Inc., Cary, NC, USA) or SPSS® version 28 (IBM Corporation, Armonk, NY). We examined task completion and tolerance wearing the HR monitor to assess feasibility and acceptability. We utilized HRV (as measured by RMSSD) in addition to Baseline-corrected HRV by calculating the change in HRV from Baseline to Relaxation and Baseline to Breathing. Given the small sample size and preliminary nature of these data, exploratory generalized linear models were utilized to assess the relationship between HRV and clinical outcome measures (CGI-I, EDI, ABC-I, FS, and BRIEF-2 ERI) across visits. Residual plots of the outcome (dependent) variables were examined and deemed appropriate for analysis. In these models, the response was the particular outcome measure (CGI-I, EDI, ABC-I, FS, and BRIEF-2 ERI), and the independent variable was a specific HRV task (Baseline, Relaxation, and Breathing). Our sample size limited the ability to examine change in HRV in response to intervention. The repeated measures variable was visit (time) within-subject, the random effect. Covariates included visit (T5, T10, T15, and T20) and visit by HRV task interaction, as well as the demographic variables age, sex, and psychotropic medication use (yes/no). Within-subject repeated measures were modeled using the first-order auto-regressive covariance structure (AR(1)). Correction for the denominator degrees of freedom was used as described by Kenward Rogers^67^. We chose to examine both pre-intervention HRV (T5) and changes in HRV across all time points (T5, T10, T15, and T20) and their relationship to the aforementioned clinical outcome measures. Missing data were treated as random and were left as missing variables.

Additionally, for those who completed both a Screen and Baseline visit (T1 and T5), we calculated intraclass correlation coefficients (ICC) using a two-way random-effects model with absolute agreement (ICC 2, 1)^68^. The random-effects model allows for systematic differences between the two testing sessions. Further, ICC’s are better able to detect systematic differences between testing sessions than correlation coefficients^69^. If participants performed similarly across the two testing sessions, their ICC will be closer to 1. Definitive guidelines for interpreting ICC values have not been well justified; however, there are a few documented guidelines like the tiered approach suggested by Cicchetti^70^: < 0.40 = poor, 0.40–0.59 = fair, 0.60–0.74 = good, and 0.75–1.00 = excellent.

## Results

### Feasibility and acceptability

Across our full sample (n = 33), 79 HRV data collection points were successfully obtained, including those excluded (n = 3) from the final analyses due to technical or synchronization issues. Within the analyzable sample (n = 30), 54 HRV data collection points were successfully obtained. In this context, wireless cardiovascular monitoring demonstrated very high feasibility, as indicated by 100% tolerance and 100% data collection success at all attempted visits.

### Exploratory relationships between pre-intervention HRV and clinical outcomes

We examined the relationship between the three HRV tasks (Baseline, Relaxation, and Breathing) at T5 and participant clinical outcome measures (CGI-I, EDI, ABC-I, FS, and BRIEF-2 ERI) across time (T5, T10, T15, T20) while controlling for age and sex. Baseline-corrected HRV data were utilized for the Breathing and Relaxation tasks.

#### Baseline

No significant interactions were found between visit and pre-intervention Baseline HRV for the clinical outcome measures (*ps* > 0.115). Within these models, there were no significant main effects of sex (*ps* > 0.308) or medication use (*ps* > 0.216). A significant effect of age was found for the FS Total scale, *F*(1, 29.71) = 6.06, *p* = 0.020.

#### Relaxation

No significant interactions were found between visit and pre-intervention Relaxation HRV for any of the clinical outcome measures (*ps* > 0.067). Within these models, there were no significant effects of age (*ps* > 0.056), medication (*ps* > 0.266), or sex (*ps* > 0.309).

#### Breathing

A statistically significant interaction between pre-intervention Breathing HRV and time was found for the EDI-D, *F*(3, 52.81) = 4.76, *p* = 0.005. Specifically, as pre-intervention Breathing HRV increases, EDI-D scores decrease at T20 (β = − 0.93, *SE* = 0.31, *t* = − 2.97, *p* = 0.005). Similarly, a significant interaction between pre-intervention Breathing HRV and time was found for the FS Social Flexibility, *F*(3, 54.93) = 7.15, *p* = 0.001, Transitions/Change, *F*(3, 54.75) = 5.97, *p* = 0.001, and Total Score scales, *F*(3, 51.89) = 5.12, *p* = 0.004. Specifically, as pre-intervention Breathing HRV increases, FS Social Flexibility (β = − 4.11, *SE* = 1.16, *t* = − 3.54, *p* = 0.001), Transitions/Change (β = − 8.62, *SE* = 2.35, *t* = − 3.66, *p* = 0.001), and Total Score (β = − 14.83, *SE* = 5.12, *t* = − 2.89, *p* = 0.006) scales decrease at T20. There were no main effects across the T10 or T15 visits (*ps* > 0.262). There were no statistically significant interactions found between pre-intervention Breathing HRV and time for the ABC-I, EDI-R, CGI, and BRIEF-2 ERI scales (*ps* > 0.145). Within these models, there were no significant main effects of sex (*ps* > 0.462) or medication use (*ps* > 0.190). A significant effect of age was found for the FS Total scale, *F*(1, 26.93) = 5.72, *p* = 0.024.

### Exploratory relationships between changes in HRV and clinical outcomes

We examined the relationship between changes in the three HRV tasks (Baseline, Relaxation, and Breathing) and participant clinical outcome measures (CGI-I, EDI, ABC-I, FS, and BRIEF-2 ERI) across time (T5, T10, T15, T20) while controlling for age and sex. Baseline-corrected HRV data were utilized for the Breathing and Relaxation tasks.

#### Baseline

A statistically significant interaction between changes in Baseline HRV and time was found for EDI-R, *F*(3, 8.14) = 5.95, *p* = 0.019. Specifically, as Baseline HRV increases, EDI-R scores decrease at T15 (β = − 5.97, *SE* = 2.25, *t* = − 2.65, *p* = 0.030) and increase at T20 (β = 1.93 *SE* = 0.71, *t* = 2.72, *p* = 0.027). There were no statistically significant interactions found between changes in Baseline HRV and time for the ABC-2, CGI, FS, and BRIEF-2 ERI scales (*ps* > 0.101). Within these models, there were no significant main effects of sex (*ps* > 0.796), medication use (*ps* > 0.151), and age (*ps* > 0.064).

#### Relaxation

There was no statistically significant interaction between changes in Relaxation HRV and time for the FS Transitions/Change subscale, *F*(3, 29.14) = 2.84, *p* = 0.055. However, as Relaxation HRV increases, FS Transitions/Change scores decrease at T20 (β = − 22.08, *SE* = 10.79, *t* = − 2.05, *p* = 0.049). No statistically significant interactions were found between changes in Relaxation HRV and time for the ABC-I, EDI-R, CGI, FS Social Flexibility, FS Total, and BRIEF-2 ERI scales (*ps* > 0.094). Within these models, there were no significant main effects of sex (*ps* > 0.584), medication use (*ps* > 0.317), or age (*ps* > 0.070).

#### Breathing

No significant interactions were found between visit and changes in Breathing HRV for the clinical outcome measures (*ps* > 0.222). Within these models, there were no significant effects of sex (*ps* > 0.379), medication use (*ps* > 0.212), or age (*ps* > 0.010).

### Test–retest reliability of HRV

Test–retest reliability was examined using ICCs between T1 and T5 for participants who completed both time points (n = 9). Overall, we found good to fair reliability across the three HRV tasks. Specifically, the Baseline task had an ICC of 0.72 (95% CI 0.13–0.93), the Relaxation task 0.62 (95% CI − 0.1–0.90), and the Breathing task 0.55 (95% CI − 0.18–0.88).

## Discussion

Several characteristics associated with ASD can predispose individuals to ED^2,4,5^, with ED being related to poorer outcomes in autistic individuals compared to those with autism without ED^6–9^. Due to the limitations in assessing ER using parent- and self-reported measures^10^, researchers have advocated for biological or physiological methods to assess ER in ASD^11^. The ANS provides a promising lens to objectively measure emotional functioning, given it is one of the body’s primary means of regulating physiological arousal, especially in autistic individuals who may not be able to communicate how their body is feeling or responding. To the authors' knowledge, our pilot study is one of the first to provide promising results on the feasibility, utility, and construct validity with clinical measures using HRV within an intervention targeting ED-specific symptoms in ASD.

Regarding feasibility, all 33 participants from the pilot trial were able to contribute HR data across multiple tasks and time points with minimal data loss, resulting in 100% data collection, including those with data that were not analyzable due to technical or synchronization issues. Further, all participants tolerated wearing the HR device with ease. Surprisingly, while we overprepared to support participants in completing the HRV task, our participants required very minimal behavioral prep outside of providing visuals, modeling, and exposure to the HRV device. Our success with collection and tolerability within this pilot study builds on the support for using HRV in interventions targeting ED in ASD and may also be a useful tool for other behavioral intervention work in ASD^47^.

The present study also examined test–retest reliability across the three different HRV tasks (Baseline, Breathing, and Relaxation) during the two pre-intervention visits (T1 and T5). Test–retest reliability considers the variability between individuals’ repeated measurements relative to the overall group variance^71^. Importantly, HRV has demonstrated high test–retest reliability in typically developing children and adolescents; however, reliability has been found to decrease in a heterogeneous sample of children with medical illnesses or conditions^72^. Overall, our reliability estimates measured in the good range for both the Baseline and Relaxation tasks (ICC’s ≥ 0.62), with fair reliability during the Breathing task (ICC = 0.55)^70^. The reliability estimates found for Baseline HRV mirror those found in autistic preschoolers^73^. Since changes in HRV are an index of physiological responses, greater reliability estimates during the Baseline and Relaxation tasks suggest minimal physiological changes were observed. However, the lower reliability estimates observed during the structured Breathing task provide promising evidence of physiological flexibility while engaged in deep or structured breathing activities, which has meaningful clinical implications. Specifically, there are likely intra-individual differences in physiological regulation in autistic youth, building on the promise of HRV as a biomarker for potential treatment response.

Additionally, the present study demonstrated construct validity with several clinical measures that may help delineate treatment outcomes for a subset of individuals. Specifically, autistic youth who had increased, or better, pre-intervention HRV (T5) during the Breathing task demonstrated improved caregiver-reported scores on the EDI-D along with increased flexibility skills (FS Total, Social Flexibility, and Transitions/Change scales) 10 weeks posttreatment. This suggests individuals with ASD + ED who begin treatment with greater PNS control during a structured breathing task may be more likely to exhibit long-term treatment gains related to ER, specifically in the areas of behavioral flexibility and dysphoria. These findings align with our previous work^52^, suggesting a performance-based behavioral measure of cognitive flexibility being sensitive to treatment change within the Regulating Together intervention. Pre-intervention HRV for Baseline and Relaxation were not related to any clinical outcomes. Although this finding was inconsistent with our prediction and suggests that our baseline and unstructured relaxation tasks may not be as good at identifying potential treatment responders, similar to the findings from Baker et al.^46^, we are cautious in this interpretation given the relatively small sample size of our pilot study.

Interestingly, while the Breathing task may not have ideal test–retest reliability, making it potentially unsuitable as a biomarker for ASD compared to their baseline or relaxation state, this task was predictive of treatment outcomes. Rather, the heterogeneity within a structured breathing task could inform who is likely to best respond to ER treatment. Additional work with larger and more diverse samples is needed to explore these findings further. With the known heterogeneity of ASD^74^, precision medicine methodology is critical for moving the treatment field forward.

Further, our results suggest increased Baseline HRV across treatment time points related to reduced caregiver-rated reactivity on the EDI-R at 5 weeks posttreatment (T15) but increased reactivity at 10 weeks (T20). Given our pilot treatment results demonstrated a significant reduction of reactivity on the EDI-R across all posttreatment timepoints^49^, it is difficult to reconcile this seemingly disparate finding. One possible explanation is that our HRV data represents a subset of our larger treatment group and uniquely captures a phenomenon in this smaller group. Alternatively, since we did not see pre-intervention Baseline HRV relating to any clinical outcomes, it is possible that the unstructured nature of the baseline task makes it less clinically meaningful^46^.

Although our findings are promising for future work, there are limitations to consider when interpreting these results. First, our sample was limited demographically, with most participants being White and Non-Hispanic. Future ASD intervention research should consider examining differences in race and ethnicity in relation to HRV and clinical outcomes, given previous work suggesting HRV was predicted by race^75^. Further, our sample size was smaller than anticipated due to data collection being halted for a period during COVID-19, with our post-intervention collection points being the most impacted. Consequently, future studies should consider recruiting larger, more diverse populations to understand better any racial or ethnic differences along with co-occurring ADHD with respect to HRV and ED. Additionally, our post-treatment collection time points were limited to 10 weeks after completion of the intervention. It would be interesting to see if HRV is predictive of or related to clinical outcomes beyond 10 weeks. Our results also did not control for autism symptom severity. This should be included in future work given recent findings suggesting reduced HRV is associated with increased autism symptomology^76^. Lastly, while our study focused on HRV during the use of positive coping tools (breathing and relaxation), future work could consider examining HRV within tasks that induce ED. In sum, the need for objective biomarkers remains when examining the impact of ER treatment in ASD. Hopefully, future work will build on our preliminary findings and consider combining multiple biomarkers to guide ER treatment development and outcomes effectively.

Taken together, our findings support the feasibility, reliability, and construct validity of HRV when utilized in a treatment trial targeting ER in ASD. While adding HRV to the Regulating Together trial^49^ was to pilot feasibility initially, we believe the results reported can be useful for planning future studies. Our findings suggest HRV is a good directly observable outcome measure that compliments clinician- and caregiver-reported measures. Although preliminary, our findings point to certain HRV profiles potentially being predictive of long-term outcomes. Clinically, HRV data could help answer important questions such as identifying individuals most likely to respond well to ER treatment and the impact on long-term treatment outcomes. HRV may also be a useful, objective tool in determining individuals needing additional long-term follow-up care to maintain intervention results at the screening or baseline stage.

## Data Availability

The datasets analyzed for this study can be requested by contacting the corresponding author, Debra Reisinger.
